# Opportunities for predictive proteogenomic biomarkers of drug treatment sensitivity in epithelial ovarian cancer

**DOI:** 10.3389/fonc.2024.1503107

**Published:** 2025-01-07

**Authors:** Trudy J. Philips, Britt K. Erickson, Stefani N. Thomas

**Affiliations:** ^1^ Molecular Pharmacology and Therapeutics Graduate Program, University of Minnesota School of Medicine, Minneapolis, MN, United States; ^2^ Department of Obstetrics, Gynecology and Women’s Health, University of Minnesota School of Medicine, Minneapolis, MN, United States; ^3^ Department of Laboratory Medicine and Pathology, University of Minnesota School of Medicine, Minneapolis, MN, United States

**Keywords:** ovarian cancer, PARP inhibitor, proteogenomics, immunotherapy, biomarkers, antibody drug conjugate (ADC)

## Abstract

Genomic analysis has played a significant role in the identification of driver mutations that are linked to disease progression and response to drug treatment in ovarian cancer. A prominent example is the stratification of epithelial ovarian cancer (EOC) patients with homologous recombination deficiency (HRD) characterized by mutations in DNA damage repair genes such as *BRCA1/2* for treatment with PARP inhibitors. However, recent studies have shown that some epithelial ovarian tumors respond to PARP inhibitors irrespective of their HRD or *BRCA* mutation status. An exclusive focus on the genome overlooks the significant insight that can be gained from other biological analytes, including proteins, which carry out cellular functions. Proteogenomics is the integration of genomics, transcriptomics, epigenomics and proteomics data. This review paper provides novel insight into the role of proteogenomics as an analytical approach to identify predictive biomarkers of drug treatment response in epithelial ovarian cancer. Proteogenomic analysis can facilitate the identification of predictive biomarkers of drug treatment response, consequently greatly improving the stratification of patients with EOC for treatment towards a goal of personalized medicine.

## 1 Ovarian cancer pathology

Ovarian cancer is the second deadliest gynecological cancer globally with a 46% 5-year survival rate (1, 2, 5–7). In 2024, there will be an estimated 19,680 new cases of ovarian cancer and 12,740 ovarian cancer-related deaths in the United States (3). More than 70% of epithelial ovarian cancers (EOCs) are of the high grade serous (HGSOC) subtype (4).

The majority of HGSOC cases are diagnosed at an advanced stage due to the absence of specific symptoms and the lack of effective screening tests (5–7). Pelvic examination, positron-emission tomography, transvaginal ultrasound, magnetic resonance imaging (MRI), laparoscopy, and CA125 and HE4 protein biomarker levels measured using enzyme linked immunosorbent assay (ELISA) are utilized as screening tests for EOC (8–10). However, the sensitivity and specificity of these diagnostic tests do not reach the targets of >75% and 99.7%, respectively to achieve a positive predictive value (PPV) of 10% (11).

## 2 Current treatment modalities

The standard treatment for EOC is cytoreductive (debulking) surgery followed by a combination of platinum (cisplatin and carboplatin) and taxane (paclitaxel or docetaxel) based chemotherapy (3, 12–15). Most HGSOCs exhibit an initial high chemosensitivity; however, ~75% of patients relapse within 5 years after first-line treatment, resulting in a low 5-year survival rate of <50% (13, 15, 16). Other EOC subtypes, such as mucinous and clear cell, are highly resistant to chemotherapy. This increases the need for novel treatments with enhanced efficacy, such as targeted therapy and immunotherapy. Three classes of targeted therapies are currently used for the treatment of EOCs: anti-angiogenic agents, poly (ADP-ribose) polymerase inhibitors (PARPi) and antibody drug conjugates.

### 2.1 Targeted therapy: anti-angiogenesis drugs

In June 2018, the humanized monoclonal antibody against vascular endothelial growth factor (VEGF), bevacizumab (Avastin), became the first US FDA-approved antiangiogenic drug for the treatment of stage III and IV EOC after surgery (17–19). Bevacizumab binds to circulating VEGF-A to competitively prevent it from binding to its endothelial cell surface receptor (VEGFR), thereby inhibiting tumor angiogenesis (20).

National Comprehensive Cancer Network (NCCN) guidelines allow for the combination of bevacizumab in multiple clinical scenarios including combination with chemotherapy as primary adjuvant therapy, platinum-sensitive relapse, and platinum-resistant EOC. Additionally, bevacizumab can be given alone for platinum resistant disease (18). Combining chemotherapy with bevacizumab increases progression free survival (PFS) (20). Other antiangiogenic drugs such as aflibercept, anlotinib and cediranib are being investigated in clinical trials for the management of EOC (21–24).

### 2.2 Targeted therapy: PARP inhibitors

Poly (ADP-ribose) polymerase (PARP) proteins are a group of 17 glycosyl-transferase nuclear enzymes with roles in DNA repair via an enzymatic process termed PARylation (25, 26). As part of the DNA damage response, *BRCA1* and *2* function within the homologous recombination repair (HRR) pathway. When these genes are mutated, DNA can be repaired via alternative pathways that involve PARP. Inhibition of PARP in homologous recombinant (HR) deficient cancer prevents DNA damage repair, resulting in cell death through synthetic lethality (18, 27–29).

Olaparib, niraparib and rucaparib are the three PARP inhibitors (PARPi) approved by the FDA for the treatment of advanced germline *BRCA*-mutated ovarian cancer. These PARPi are also approved for use as maintenance therapy for EOC. In 2014, olaparib became the first PARPi targeting PARPs 1-4 approved by the FDA as a monotherapy for the treatment of advanced germline *BRCA*-deficient ovarian cancer, and it was approved in 2017 as maintenance therapy for platinum-sensitive EOC (SOLO-1 and SOLO-2 clinical trials, respectively) (20, 30–36). Rucaparib targets most of the PARP enzymes (PARPs 1-4, 12, 15 and 16). In 2016, it became the second PARPi to receive FDA approval as maintenance therapy for advanced ovarian cancer (20, 30), and it is currently approved for the treatment of BRCAmut recurrent EOC based on data from the ARIEL 3 and 4 clinical trials (25, 37–39) Niraparib is another PARP1/2 inhibitor with FDA approval as maintenance therapy for recurrent EOC. Currently, niraparib is approved as maintenance therapy for platinum-sensitive recurrent ovarian cancer independent of *BRCA1/2* mutation and HR status based on results from the PRIMA and NOVA clinical trials (40–43). Emerging PARPi under clinical investigation for the treatment of EOC include talozaparib, veliparib, pamiparib and fuzuloparib (44, 45, 62). **Table 1** lists the clinical trials associated with the currently approved and emerging PARPi.

**Table 1:** Clinical trials of PARPi alone or in combination with anti-angiogenic drugs and/or chemotherapy.

Clinical trial identifier (Trial name)	Phase	Status	Study population and numbers	Treatment	Outcome
NCT01844986 (SOLO-1)	III	Active, not recruiting (Approved)	391 patients with BRCAmut ovarian cancer following first-line platinum based chemotherapy	Olaparib (maintenance therapy)	Mean PFS for olaparib 36.39 months vs. 21.46 months for placebo, p<0.0001 (33)
NCT03402841 (OPINION)	IIIb	Completed	279 non-germline BRCAmut platinum-sensitive HGSOC	Olaparib	Median PFS 16.4, 11.1, 9.7 and 7.3 months for sBRCAmut, HRD-positive with sBRCAmut, HRD-positive without sBRCAmut and HRD-negative patients, respectively (114)
NCT02477644 (PAOLA-1)	III	Completed	806 patients with advanced FIGO stage IIIB - IV HGSOC or endometrioid ovarian, fallopian tube, or peritoneal cancer treated with standard first-line treatment; platinum-based chemotherapy plus bevacizumab	Olaparib Bevacizumab Placebo	PFS 22.1 months for olaparib + bevacizumab vs. PFS 16.6 months for placebo + bevacizumab (35)
NCT06121401 (IOLANTHE)	IV	Active, recruiting	190 patients with advanced stage HRD positive HGSOC	Olaparib plus Bevacizumab as first-line treatment	ongoing
NCT02446600 (NRG-GY004)	III	Active, not recruiting	579 women with recurrent platinum-sensitive HGSOC, primary peritoneal or fallopian tube cancer	Platinum-based chemotherapy (carboplatin, paclitaxel, pegylated liposomal doxorubicin hydrochloride) Olaparib (alone) Cediranib plus Olaparib	ongoing
NCT02502266	II & III	Active, not recruiting	587 patients with recurrent platinum-resistant or refractory HGSOC, primary peritoneal or fallopian tube cancer	Cediranib plus Olaparib Olaparib (alone) Cediranib Chemotherapy (Paclitaxel, Topotecan)	ongoing
NCT02345265	II	Active, not recruiting	70 patients with recurrent platinum-resistant or platinum-sensitive ovarian cancer	Cediranib plus Olaparib	ongoing
NCT01891344 (ARIEL2)	II	Completed	287 patients with platinum-sensitive, relapsed HGSOC, fallopian tube or primary peritoneal cancer	Rucaparib	Median PFS 12.8 months for BRCAmut patients vs. 5.7 months for BRCAwt patients (115)
NCT01968213 (ARIEL3)	III	Completed	564 patients with recurrent platinum-sensitive HGSOC, fallopian tube or primary peritoneal cancer	Rucaparib	Median PFS 16.6 months for rucaparib vs. 5.4 months for placebo (116)
NCT02855944	III	Completed	349 patients with relapsed ovarian cancer (BRCA1 or BRCA2 mutation)	Rucaparib Platinum-based chemotherapy (paclitaxel/cisplatin/carboplatin)	Median PFS 7.4 months for rucaparib vs. 5.7 months for chemotherapy (37)
NCT01847274 (ENGOT-OV16/NOVA study)	III	Completed	553 patients with platinum-sensitive HGSOC	Niraparib	gBRCAmut: Median PFS 21 months vs. 5.5 months for placebo; non-gBRCAmut: Median PFS 12.9 months for niraparib vs. 3.8 months for placebo (117)
NCT02655016 (PRIMA)	III	Active, not recruiting	733 patients with advanced stage HGSOC following first-line platinum-based chemotherapy	Niraparib	ongoing
NCT02470585	III	Completed	1140 patients with advanced stage HGSOC, fallopian tube and primary peritoneal cancer	Veliparib plus Carboplatin and Paclitaxel	Median PFS 34.7 months for veliparib vs. 22.0 months for control (118)
NCT05489926 (PamiAP)	II	Active, not recruiting	15 patients with EOC and prior exposure to PARPi	Pamiparib	

### 2.3 Targeted therapy: antibody drug conjugates

Antibody drug conjugates (ADC) consist of a cytotoxic drug conjugated through a linker to a monoclonal antibody targeting specific tumor-associated antigens. ADCs use monoclonal antibodies to deliver cytotoxic drugs to cancer cells by selectively binding to a specific antigen (typically a cell surface receptor) overexpressed on the surface of cancer cells. ADCs have shown potential positive effects in recurrent and platinum-resistant ovarian cancer. Mirvetuximab soravtansine (MIRV) is the only FDA approved ADC for the treatment of folate receptor alpha (FRα) positive platinum-resistant epithelial ovarian cancer (46–50). It is comprised of an anti-FRα monoclonal antibody, a cytotoxic payload (maytansinoid, a microtubule inhibitor) and a cleavable disulfide linker. The SORAYA (51) and MIRASOL (52) trials demonstrated the efficacy of MIRV in platinum-resistant ovarian cancer with an increase in PFS when compared to chemotherapy alone. Ongoing trials for the treatment of platinum-resistant ovarian cancer include the use of other ADCs such as Farletuzumab ecteribulin (53), Raludotatug deruxtecan (54), Luveltamab tazevibulin (55), Anetumab ravtansine (56, 57) and Lifastuzumab vedotin (58).

### 2.4 Targeted therapy: immunotherapy

EOC tumors are “immunogenic tumors” that cause sporadic anti-tumor immune responses detected in the peripheral blood, ascites and tumors (59, 60). The tumor microenvironment (TME) is implicated in EOC prognosis (61, 62). Early studies demonstrated evidence of tumor-infiltrating lymphocytes (TILs), programmed death 1 (PD-1) and programmed death-ligand 1 (PD-L1) in EOC. TILs, PD-1 and PD-L1 correlate with improved EOC prognosis, which renders immunotherapy for ovarian cancer an area of great interest (60, 63). Some immunotherapies for EOC include immune checkpoint inhibitors (ICI), chimeric antigen receptor T (CAR-T) cells, vaccines and monoclonal antibodies (62). Nivolumab and Pembrolizumab are ICIs for PD-1 that have been tested for the treatment of relapsed platinum-resistant and advanced ovarian cancer, respectively. Avelumab, an anti-PD-L1 antibody, and Ipilimumab, an anti-CTLA-4 antibody, have been tested for the treatment of recurrent platinum-sensitive ovarian cancer (60, 63). Despite a justified rationale for the use of ICIs in EOC, the results from clinical trials have shown a limited clinical efficacy of ICIs; the median overall response rates (ORR) are 10-15% (64, 65). An ORR of 22.2% was achieved in a study of 12 patients with recurrent or metastatic EOC treated with the anti-PD-L1 inhibitor, Atezolizumab (65, 66). These mediocre ORRs are complicated by several adverse events experienced by up to 75% of patients, including fatigue, gastrointestinal, endocrine and dermatological events, and neurological, cardiological, pulmonary and renal toxicities (67, 68). The limited efficacy of immune checkpoint blockade in the setting of EOC could be due to several factors, including the compensatory upregulation of alternative immune checkpoints, low tumor mutational burden, loss of mutation-associated antigens, or the expression of multiple inhibitory receptors on the infiltrating T-cells (60, 61, 64, 66).

Additionally, immunotherapy has been added to chemotherapy in multiple large phase III trials of patients with primary and recurrent ovarian cancer. Unfortunately, across all of these studies, patients who received immunotherapy did not derive any benefit in terms of PFS and OS compared to those who received placebo. It is not known if there may be subsets within this population that could derive some benefit from immunotherapy — this is an area in need of further evaluation.

Several clinical trials are ongoing for ICIs combined with platinum-based chemotherapy, PARPi, anti-angiogenic agents and other biologic agents in recurrent EOC (**Table 2**). Of note, immunotherapy is not currently incorporated in routine clinical practice.

**Table 2:** Clinical trials of immune checkpoint inhibitors combined with PARPi or anti-angiogenic drugs and/or chemotherapy.

Clinical trial identifier (Trial name)	Phase	Status	Study population and numbers	Treatment	Outcome
NCT05158062 (SaINT-ov02)	II	Recruiting	35 patients with platinum-sensitive recurrent ovarian cancer	Pembrolizumab Bevacizumab plus chemotherapy (carboplatin, paclitaxel, docetaxel) Olaparib	ongoing
NCT04519151	II	Recruiting	24 patients with platinum-sensitive recurrent ovarian cancer	Pembrolizumab Lenvatinib	ongoing
NCT035744779 (OPAL)	II	Active, not recruiting	123 patients with recurrent ovarian cancer, fallopian tube or primary peritoneal cancer	TSR-042 (Dostarlimab) Niraparib Bevacizumab Chemotherapy (Carboplatin, paclitaxel)	ongoing
NCT05751629 (MOONSTONE/GOG-3032)	II	Completed	41 patients with advanced relapsed HGSOC, fallopian tube, primary peritoneal who received 1 – 2 prior lines of anticancer therapy, are PARPi naïve, and have platinum-resistant but not refractory disease	Dostarlimab Bevacizumab Niraparib	Study terminated due to adverse events (119)
NCT02953457	II	Active, not recruiting	40 patients with ovarian, fallopian tube, or primary peritoneal cancer with BRCA1 or BRCA2 mutation	Durvalumab Olaparib Tremelimumab	ongoing
NCT04034927	II	Active, not recruiting	61 patients with recurrent ovarian cancer, fallopian tube or primary peritoneal cancer	Olaparib Tremelimumab	ongoing
NCT02571725	I & II	Active, not recruiting	50 patients with recurrent BRCAmut-associated ovarian cancer	Olaparib Tremelimumab	ongoing
NCT05065021 (Re-VOLVE)	II	Recruiting	40 patients with ovarian cancer who previously received PARPi	Niraparib Dortarlimab Bevacizumab Paclitaxel	ongoing

## 3 Treatment resistance and disease recurrence

Biomarkers are measured and evaluated as an indication of normal biological processes, disease state and pharmacological response to treatment (69). They can serve as tools to screen for, characterize, or diagnose disease, consequently enabling personalized drug treatment and the prediction of drug toxicity and adverse drug reactions (5). Predictive biomarkers measure the potential response or lack of response to a particular treatment, helping to identify patients who are likely to benefit from the treatment (69).

In EOC, >80% of patients respond to the first line of treatment, i.e. cytoreductive surgery and platinum-based chemotherapy, with approximately 80% having recurrence at different time intervals after chemotherapy (70–72). Patients having recurrence >6 months of standard treatment are considered platinum-sensitive. Recurrence during chemotherapy or within a month after treatment is referred to as chemotherapy-refractory ovarian cancer (73, 74). This recurrence is intrinsic or a form of primary resistance. Identifying this primary resistance using predictive biomarkers can help patients receive more effective alternative treatments.

Another form of recurrence occurs in patients who initially respond to chemotherapy but develop recurrence within 6 months of treatment. These patients’ tumors are referred to as platinum-resistant (73, 75). This type of acquired resistance occurs after exposure to drug treatment (76). Predictive biomarkers for acquired resistance can help determine when to change treatment. Less than 20% of clear cell, mucinous, endometrioid, and LGSOC tumors respond to standard treatment. Approximately 90% of patients with HGSOC respond to standard treatment, but some patients still relapse after treatment (74). This increases the need to understand the molecular basis of EOC for improved disease management (70).

Among the factors that influence drug resistance by decreasing the intracellular drug concentration are increased expression of drug efflux pumps (e.g., copper-transporter 2, which is involved in the efflux of platinum), overexpression of ATP-binding cassette (ABC) transporters (e.g., P-glycoprotein), and the decreased expression of drug influx transporters (77). P-glycoprotein is highly expressed in EOC patients with decreased PFS, therefore drug efflux pumps or drug transporters are candidate predictive biomarkers due to their ability to modulate EOC resistance (74). Solute-like-carrier (SLC) family proteins are drug influx transporters that increase intracellular accumulation of drugs. Lower expression of SLC22A5 and SLC31A1 has been observed in patients with HGSOC, whereas their elevated levels correlate with increased treatment response and survival rates (77). Dysregulation of autophagy and evasion of apoptosis also contribute to drug resistance (77). Hence, the proteins involved in these pathways represent feasible predictive biomarker candidates.

The activation of DNA damage repair pathways also plays a major role in ovarian cancer treatment resistance. DNA damaging agents such as platinum-based chemotherapies cause double strand breaks that are repaired by the HR pathway to increase cell survival. Several academic and commercial entities have developed tests to assess the genomic instability of tumor DNA as a biomarker of HR (73, 74, 78). Deleterious mutations in the *BRCA1/2* tumor suppressor genes lead to error-prone non-homologous end joining (NHEJ), resulting in genome instability (73, 77, 79, 80). EOC patients with *BRCA1* or *BRCA2* mutations are sensitive to DNA-damaging agents. Other proteins such as RAD51, ATM, ATR and PALB2 are also involved in HR, and mutations in the genes encoding for these proteins increase the sensitivity of HGSOC patients to chemotherapy (81, 82). Most HGSOC patients with HR deficiency have an increased response rate to platinum-based therapy (83).

The combination of standard treatment with other chemotherapies or targeted therapy can help improve patient outcomes. Stratifying patients for specific drug treatments based on predictive biomarkers has the potential to increase drug response and reduce drug resistance in the context of personalized or precision medicine.

## 4 The role of predictive biomarkers in precision medicine-based treatment of EOC

Over the past decade, biomedical research has focused on understanding the disease state of individual patients to develop specific diagnosis and treatment options, forming the basis for precision medicine, which is known as personalized medicine (84). In 2015, U.S. President Barack Obama announced the launch of the Precision Medicine Initiative (PMI) to improve healthcare. The goal of the PMI was “to enable a new era of medicine through research, technology, and policies that empower patients, researchers and providers to work together toward the development of individualized care” (85, 86). The initiative focused on providing the most effective treatment for patients based on their genetic changes. The following year, the National Cancer Moonshot Initiative was launched by then Vice President Joe Biden to improve cancer prevention, diagnosis and treatment with the goal of ending cancer deaths (86, 87).

Precision medicine aims to use information from patients’ genes, proteins and the environment to identify early health indicators, detect disease stages, slow disease progression, and alter the health trajectories of patients through targeted treatments or lifestyle changes (84, 88, 89). In oncology, precision medicine has significantly improved cancer treatment by identifying the unique molecular characteristics of different cancers, allowing for the development of targeted therapies that can effectively target specific cancer types (90). An example is EOC patients with *BRCA1/2* mutations who benefit from PARPi (91). Additionally, with precision medicine, patient response to drugs can be assessed using molecular assays to test the sensitivity of the tumor cells to current or emerging therapies (92). Precision medicine leads to better treatment outcomes and reduces the trial-and-error treatment approach, which has traditionally followed a “one-size fits all” modality (93).

Predictive biomarkers play an important role in cancer precision medicine whereby they serve as tools to predict treatment response. Genome-based biomarkers were the first widely used predictive biomarkers in cancer precision medicine focusing on genome sequencing to identify cancer-specific somatic and germline mutations to provide information on cancer susceptibility and treatment options (84, 94, 95). Individuals with germline mutations in specific oncogenes are at higher risk of developing cancer. These mutations include missense mutations, nonsense mutations, insertions or deletion and rearrangements (94).

## 5 Opportunities for predictive proteogenomic biomarkers in EOC precision medicine

Despite the promising potential of genome-based predictive biomarkers in precision medicine, several limitations remain, including the complexity and variability of these mutations, which can affect the accuracy and reliability of predictive biomarkers. These mutations can vary widely even within different areas of the same tumor, making it difficult to develop an effective treatment approach based on a single biopsy.

Genomics has provided fundamental insights into the disease mechanism of several cancer types; however, genomic data alone does not predict disease prognosis and treatment efficacy due to the indirect correlation between genotypes and phenotypes (96). Transcriptomics, proteomics, epigenetics and metabolomics can be utilized as biomarker tools in cancer precision medicine (95). Another analytical approach known as proteogenomics has recently gained importance in cancer precision medicine.

Proteogenomics is the integration of genomics, transcriptomics, epigenomics and mass spectrometry-based proteomics data, which aims to provide a comprehensive view of the molecular basis of disease processes (96, 97). By identifying novel protein-coding regions, post-translational modifications (PTMs), proteoforms, and single amino acid variations, proteogenomics enables the exploration of the functional consequences of genetic variations (98).

Proteogenomic analysis entails whole-genome sequencing (WGS) or whole exome sequencing (WES) to identify genetic variants and genomic mutations, RNA-seq to identify alternative splicing and non-coding RNAs, and mass spectrometry-based proteomic analysis to identify and quantify peptides (99). The peptides generated from proteomic analysis are mapped to a custom protein sequence database derived from the sample-specific genomic, transcriptomic or epigenomic data instead of a reference proteome database to identify possible novel proteins (100). The custom protein database can be generated using bioinformatics tools such as Galaxy-P, customProBD, sapFinder and PGA (98).

Proteogenomics has been applied in cancer research in an approach termed onco-proteogenomics to identify tumor-specific peptides. Onco-proteogenomics enables the validation of the translation of specific cancer-associated genomic mutations into proteins (phenotypes), and the identification of PTMs and altered signaling pathways involved in cancer development (98). The National Cancer Institute’s Clinical Proteomic Tumor Analysis Consortium (CPTAC) has published several landmark studies applying proteogenomics to characterize the molecular landscape of multiple cancer types, including breast, colorectal, pancreatic, lung, clear cell renal, and ovarian carcinoma (101–104). Onco-proteogenomics has a role in the identification and development of diagnostic, prognostic, and predictive biomarkers.

Liquid biopsies containing circulating tumor cells (CTCs) can be utilized as samples for onco-proteogenomic testing. CTCs contain molecular markers from patient tumors, which can be measured to assess drug treatment response (105). Given that the somatic mutational landscape of HGSOC remains stable with chemotherapy treatment, subjecting patients to rebiopsy at each recurrence time point would not be informative or practical due to patient discomfort and the financial cost (105, 106). In contrast, it is possible that the somatic proteogenomic landscape of HGSOC is altered during the course of chemotherapy treatment. Establishing proteogenomic biomarkers of treatment response using CTCs represents a novel, feasible, and minimally invasive approach.

Most molecularly targeted therapies target proteins in cancer cells. Examples of these targeted therapies include PARP inhibitors, kinase inhibitors and immunomodulatory proteins (102). Proteogenomics can help identify predictive biomarkers for the diagnosis and effective treatment of patients with EOC. PARPi are particularly effective in EOC patients with *BRCA1/2* mutations or HRD. Proteogenomic analyses can help identify additional biomarkers beyond *BRCA* mutations, such as specific protein expression patterns or mutations in other DNA repair genes that can predict sensitivity to PARP inhibitors (107). This could expand the population of EOC patients who benefit from these therapies.

In ovarian cancer, proteogenomics has identified PTMs such as phosphorylation, acetylation and glycosylation as candidate predictive biomarkers of response to targeted therapies. A CPTAC study integrating the proteomic measurements of 174 HGSOC tumor samples with the genomic data generated by TCGA provided several novel insights into HGSOC biology, including the association between protein relative abundance and chromosomal instability, the impact of copy number alterations (CNA) on the proteome, the signaling pathways that diverse genome rearrangements coverage on, and the signaling pathways that are associated with short overall survival (108, 109). The proteogenomic and phosphoproteomic characterization of 83 HGSOC and normal fallopian tube tissues identified signaling pathways that differentiated HGSOC from normal tissues based on their HRD status (109, 110). These studies also demonstrated that decreased acetylation levels of Histone H4 Lysine12 and Lysine16 were associated with HRD in HGSOC (109, 110). These PTMs are candidate biomarkers for HRD, which creates the potential for HGSOC patients to be stratified for treatment with DNA repair pathway targeted therapies, including histone deacetylation (HDAC) inhibitors and PARPi (108, 110).

Resistance to platinum-based chemotherapy is a major challenge in EOC treatment. Proteogenomic studies can identify proteins and pathways associated with chemoresistance, such as alterations in the PI3K/AKT pathway, which could serve as predictive biomarkers (108). Proteogenomics also has potential utility in identifying predictive biomarkers of response to treatment with other targeted therapies that are used in the treatment of EOC. For example, predictive biomarkers such as PD-L1 expression, tumor infiltrating lymphocytes (CD8+, CD4+), tumor mutational burden and chemokines (CXCL 9,11,13) can be used to identify EOC patients that do or do not respond to ICI (111). Increased levels of chemokines and tumor infiltrating lymphocytes have been associated with increased response to immunotherapy (112, 113).

## 6 Conclusion and outlook

The proteogenomic molecular data from liquid biopsies of patients with EOC is a largely unexplored source of predictive biomarkers of treatment response. These samples can be obtained from patients before, during and after treatment to inform treatment decisions. CTCs contain information from the RNA, DNA, proteins and metabolites from the patient tumors and can therefore serve as valuable biospecimens for disease detection and monitoring treatment response.

*In vitro* diagnostic clinical tests can be developed and validated to measure these proteogenomic biomarkers with an intended use of predicting treatment response. This will enable patients to be stratified for treatment, consequently preventing them from being prescribed drug treatment regimens from which they could potentially not derive any benefit, which is incongruent with the goal of precision medicine. Proteogenomics is a valuable tool supporting personalized medicine efforts. Incorporating this powerful analytical method into translational research studies advances a goal of improving the overall survival of patients with EOC.
